# Muscle Asymmetries in the Lower Limbs of Male Soccer Players: Preliminary Findings on the Association between Countermovement Jump and Tensiomyography

**DOI:** 10.3390/sports10110177

**Published:** 2022-11-14

**Authors:** Alex Buoite Stella, Annalisa Galimi, Miriam Martini, Luca Di Lenarda, Luigi Murena, Manuela Deodato

**Affiliations:** 1 School of Physiotherapy, Department of Medicine, Surgery and Health Sciences, University of Trieste, Via Pascoli 31, 34100 Trieste, Italy; 2 Clinical, Integrative and Translational Physiology, Department of Medicine, Surgery and Health Sciences, University of Trieste, Strada di Fiume 447, 34149 Trieste, Italy; 3 Orthopaedics and Traumatology Unit, Cattinara Hospital-ASUGI, Department of Medicine, Surgery and Health Sciences, University of Trieste, Strada di Fiume 447, 34149 Trieste, Italy; 4 Ph.D. Program in Neural and Cognitive Sciences, Department of Life Sciences, University of Trieste, Via Weiss 2, 34100 Trieste, Italy

**Keywords:** symmetry, power, soccer, football, skeletal muscles, injury

## Abstract

Strength and power asymmetries have been observed in different sports, including soccer. Such asymmetries, as well as the bilateral deficit (BLD), can be assessed during different tasks, static or dynamic, and with different methods and devices, in order to detect the possible different aspects, as well as the association with physical performance and injuries. The aim of this study was to investigate the association between muscle asymmetries and BLD during a countermovement jump (CMJ), and tensiomyography (TMG) parameters and asymmetries, in the lower limbs of male soccer players. A total of 23 male soccer players (18 ± 4 years) were recruited. Bilateral and unilateral CMJs were performed, and peak power (W) and height (cm) were obtained. TMG was performed on different muscles of the lower limbs, and lateral and functional symmetries were obtained. Playing position and history of injuries were collected. CMJ inter-limb symmetry was found to significantly correlate with biceps femoris (r = 0.574, *p* = 0.004) and soleus (r = 0.437, *p* = 0.037) lateral symmetry. Players in central roles presented significantly worse functional symmetry scores of the knee than defense players (−17.5%, 95% CI −31.2–−3.9; *p* = 0.10). Participants reporting a history of injury at the ankle were characterized by significantly lower functional symmetry in both the dominant (43%, 39.5–48.0 vs. 74.5%, 46.5–89.3, *p* = 0.019) and non-dominant (45%, 42.5–46.0 vs. 81.0%, 45.8–90.3, *p* = 0.024) ankle. Findings from this preliminary study suggest an association between lower-limb muscle asymmetries during a dynamic task, such as jumping, and muscle contractile properties evaluated with TMG; moreover, functional asymmetries may be present after ankle injuries. Future studies in larger samples should evaluate the presence of such asymmetries as predictors or characteristics of different muscular and joint injuries.

## 1. Introduction

Sport performance involves motor tasks that can be performed with both limbs and a single limb at a time, showing differences in force and power output. Inter-limb (or lateral) and functional asymmetries in strength, power, balance, flexibility, and electromyographic muscle activity have been considered as possible factors associated with both performance and injury risk in sports [1]. Vertical jump performance, as the countermovement jump (CMJ), can provide useful information about the elastic and explosive capacity of the lower limbs [2]. The term bilateral deficit (BLD) refers to the greater expression of force or power obtained when the right and left limb unilateral performances are added together, compared with the force or power measured during the bilateral performance [3,4]. BLD has been reported in different motor tasks, such as isokinetic [5], isometric [6], and dynamic contraction types [7], and in specific sport-related motor tasks; in particular, vertical jumps have often been considered to evaluate inter-limb asymmetries and BLD in relation to different sports-specific performances [7,8,9,10,11,12]. The mechanisms that have been hypothesized to underlie the BLD include neural mechanisms such as interhemispheric inhibition [4], and mechanics, since during bilateral jumps the muscles shorten at a higher speed, resulting in a lower force output and less joint work per leg [13]. As such, due to the diversity of the contractile elements and mechanisms that seem to be involved, BLD should be considered a multifactorial phenomenon rather than dependent on a single factor [7].

Tensiomyography (TMG) is a well-validated technique developed to assess skeletal muscle contractile properties in humans non-invasively by obtaining a displacement–time curve through a sensor with a certain pre-tension on the muscle belly [14,15,16,17]. TMG assessment is independent of motivation or volitional effort, therefore representing an objective evaluation of muscle contractile properties [17]. Through the displacement–time parameters, TMG has been suggested to estimate predominant skeletal muscle fibers non-invasively [18], monitor muscle fatigue [19,20], suggest training and rehabilitation-induced adaptations [21,22,23,24,25,26], and to assess neuromuscular risk factors of ACL injury [27,28]. Previous studies have not found significant asymmetries in TMG-derived parameters in the lower limbs of non-previously injured male [26,29,30] and female soccer players [17], although TMG has been shown to be a valuable tool for assessing neuromuscular risk factors in ACL injuries [27]. As such, TMG assessments have been recommended to be performed with other measures of neuromuscular function [17].

Lateral symmetry could be assessed through jump performance and TMG, and each method could provide information about different aspects of muscle performance [30]. Since CMJ and TMG could represent different aspects of muscle contractile characteristics [31], considering both assessments could provide a better insight into muscle properties in different sports, such as soccer. Previous investigations have aimed to show associations between jump performance and TMG, but parameters have not shown significant associations with jump performance [30,31]. Nevertheless, despite the evaluation of muscle asymmetries having been proposed in different sports and with different techniques [32,33], there is not a consensus regarding the associations between BLD and jump asymmetries [34], and TMG asymmetries.

Given the above-mentioned neuromuscular characteristics of the inter-limb asymmetry and BLD, the primary aim of this study was to assess the potential associations between bilateral and unilateral jumping performance and TMG parameters. Secondly, such parameters were compared across different playing positions. Lastly, inter-limb asymmetry, BLD, and TMG asymmetries were tested as risk factors associated with players’ reported injury history.

## 2. Materials and Methods

A cross-sectional observational study was performed to assess neuromuscular responses in male soccer players. Participants were recruited among local sub-elite male soccer teams during the preseason period. Inclusion criteria were age between 16 and 40 y, a soccer training history of not less than 5 y, and a training frequency of not less than 3 times per week. Exclusion criteria included the presence of current lower-limb injuries, and frequent (more than one time per week) training in sports other than soccer. All participants were recruited and tested before the start of the 2022–2023 season of competitions. One week before data collection players were informed about the measurement procedures and detailed study protocol, and they were asked to sign the informed consent. In the case of minors, the approval of the parents or legal representative was requested. Participants were advised not to have a strenuous workout for at least 48 h before the assessment. All players were physically healthy, without acute pain, and free from serious lower-limb injury for at least one year. This study was approved by the Ethical Committee of the University of Trieste.

### 2.1. Anamnestic Questionnaire and Anthropometrics

All the participants were asked to complete a questionnaire to report demographic characteristics (body mass, body height, and ethnicity), training history and characteristics (frequency, times/week; training duration, min; and training volume, min/week), playing position (goalkeeper, defense, center, or forward), and whether they normally performed training sessions dedicated to strength and conditioning. Leg dominance was determined from the self-reported preferred kicking leg [35]. The reported history of previous injuries was revised by an expert physiotherapist with specific experience in sports injuries and rehabilitation. Muscle or joint injuries in the last 5 years were grouped into the following categories according to the involved muscles and joints: quadriceps, hamstring, knee, leg, and ankle.

### 2.2. Jump Performance, Inter-Limb Asymmetry, and BLD Assessment

After a 5-min warm-up, jumps were performed on an integrated force platform—video analysis system (D-Wall, Tecnobody, Dalmine – Bergamo, Italy), and parameters such as peak power and jump height were extracted by the software. The sampling rate of the force platform was 100 Hz, 150 g resolution, while the 3D camera collected the video at 30 fps. The video analysis supported the standardization of the positioning of the subjects before and during the jump, while the force platform collected the jump parameters. Data were then reported for each jump showing peak values that could be double-checked by comparing the force platform data with the video analysis. Time was given to familiarize the participants with procedures and jump performances. Vertical countermovement jumps (CMJs) were performed bilaterally and unilaterally on both single legs in a randomized order, with the hands on the hips. All jumps were performed three times and the higher value was considered for the final analysis, according to previous literature [7,30]. Between each jump, a 1-min rest was allowed to minimize the effect of fatigue on performance [12]. Inter-limb asymmetry was calculated as the difference between the two lower limbs’ unilateral jumps, over the limb with the higher values in the unilateral jump. BLD was obtained by the following formula for both power and jump height [36]:

$$\mathrm{BLD}(\%)=[100\times \frac{\mathrm{bilateral}}{\left(\mathrm{right}\mathrm{unilateral}+\mathrm{left}\mathrm{unilateral}\right)}]-100$$

### 2.3. Tensiomyography

TMG measurements were performed during electrically evoked maximal isometric contractions on selected skeletal muscles of the lower limbs, bilaterally, according to previously described procedures [17]. Assessed muscles were: adductor longus (m.AL), biceps femoris (m.BF), gastrocnemius lateralis (m.GL), gastrocnemius medialis (m.GM), gluteus major (m.GT), rectus femoris (m.RF), soleus (m.SO), tibialis anterior (m.TA), vastus lateralis (m.VL), and vastus medialis (m.VM). A single, 1 ms maximal monophasic electrical impulse was used to elicit a twitch contraction that caused the muscle belly to oscillate. These oscillations were recorded using a sensitive digital displacement sensor (TMG-BMC Ltd., Ljubljana, Slovenia) placed on the skin’s surface at the measuring site of the muscle of interest. Initially, the stimulation amplitude was set just above the threshold and then gradually increased until the amplitude of the radial twitch Dm (in millimeters) increased no further. Electrical pulses ranged between 85 and 110 mA at constant 30 V. An inter-stimulation time interval of 10–15 s was used. From two maximal responses, all contractile parameters were estimated and average values were taken for further consideration. The TMG parameters were: Dm [the maximal displacement (mm)], Td [delay time; the time from electrical pulse to 10% of Dm (ms)], Tc [contraction time; the time between 10% and 90% of Dm (ms)], Ts [sustain time; the time when the response was above 50% of Dm (ms)], and Tr [half-relaxation time; the time from 90% to 50% of Dm during muscle relaxation (ms)], and were extracted by TMG software (Version 3.6.16) and used for offline analysis [18,33]. Dm is the absolute spatial transverse deformation of the muscle and reduced Dm is interpreted as an increase in muscle stiffness, whereas larger Dm implies lower muscle stiffness; Td provides a measure of muscle responsiveness; Tc reflects the speed of twitch force generation, and may reflect muscle fiber type or tendon stiffness; Ts provides a theoretical assessment of muscle fiber fatigue status; and Tr is considered the least reliable parameter across studies and should be further investigated [37]. 

In addition, the TMG software applied an algorithm to calculate the lateral (i.e., inter-limb symmetry for a specific muscle) and functional symmetries (i.e., symmetry between the muscles that surround a joint) [15,17].

Lateral symmetry (LS) was defined as follows:

$$\mathrm{LS}(\%)=0.1\times (\frac{MIN\;\left(TdR;TdL\right)}{MAX\;\left(TdR;TdL\right)})+0.6\times (\frac{MIN\;\left(TcR;TcL\right)}{MAX\;\left(TcR;TcL\right)})+0.1\times (\frac{MIN\;\left(TsR;TsL\right)}{MAX\;\left(TsR;TsL\right)})+0.2\times (\frac{MIN\;\left(DmR;DmL\right)}{MAX\;\left(DmR;DmL\right)})\times 100$$

where, *MIN*—the minimum, *MAX*—the maximum, *R*—right leg parameters and *L*—left leg parameters. 

For functional symmetry (FS), different algorithms are used according to the investigated site (Achilles tendon: GL/GM; ligament patellae: *VM*/*VL*; knee: *RF*, *VL*&*VM*/*BF*; ankle: *TA*/GL&GM; leg: *VL*&*VM*/GL&GM); for the knee:

$$\mathrm{FS}(\%)=0.1\times (\frac{MIN\;\left(AVERAGE\left(TdRF;TdVL;TdVM\right);TdBF\right)}{MAX\;\left(AVERAGE\left(TdRF;TdVL;TdVM\right);TdBF\right)})+0.8\times (\frac{MIN\;\left(AVERAGE\left(TcRF;TcVL;TcVM\right);TcBF\right)}{MAX\;\left(AVERAGE\left(TcRF;TcVL;TcVM\right);TcBF\right)})+0.1\times \;(\frac{MIN\;\left(AVERAGE\left(TrRF;TrVL;TrVM\right);TrBF\right)}{MAX\;\left(AVERAGE\left(TrRF;TrVL;TrVM\right);TrBF\right)})\times 100$$

where, *MIN*—the minimum, *MAX*—the maximum, *RF*—rectus femoris, *VM*—vastus medialis, *VL*—vastus lateralis, and *BF*—biceps femoris. 

As such, LS and FS were also analyzed in this study, considering an 80% and 65% cut-off value, respectively [17].

### 2.4. Statistical Analyses

All statistical analyses were performed with SPSS v.23 (IBM Inc., Armonk, NY, USA). The Shapiro–Wilk test for normality of distribution was performed. Data are reported as mean ± standard deviation (sd) or counts and proportions (%), as appropriate. Within-subject variation (CV) was performed as a measure of reliability of the bilateral and unilateral jump performance, and TMG parameters [38,39,40]. For CMJ, CV ranged from 2.5% for bilateral height to 3.4% for non-dominant limb power. For TMG parameters, CV ranged from 2.1% for m.RF Tc in the dominant limb to 8.7% for m.AL Tr in the dominant limb. Correlations between CMJ and TMG parameters were performed and Pearson’s coefficient was reported. To test differences between playing roles, a one-way analysis of variance (ANOVA) with Bonferroni test corrections was used to identify differences in CMJ performance, symmetry, and TMG lateral symmetries; furthermore, a mixed-factors ANOVA with limb (dominant vs. non-dominant) as a within-subject factor, and playing position (defense vs. center vs. forward) as a between-subject factor, was used. Since only one goalkeeper was included in the study, they were not included in such analyses. When significant effects were found for limb or playing position (two-way interactions were excluded from the analysis), post hoc pairwise comparisons were performed for each variable independently. Partial Eta squared (pɳ2) effect size was reported for identified main and interaction effects. The criteria for effect size were small (pɳ2 = 0.01), medium (pɳ2 = 0.06), and large (pɳ2 = 0.14) [41]. Lastly, due to the limited frequency of reported injuries, a Mann–Whitney U-test was used to test differences in CMJ and TMG parameters between those reporting previous injuries in the lower limbs. Significance was set at *p* < 0.05.

## 3. Results

From twenty-five male soccer players who volunteered for the study, two participants were excluded due to the presence of ongoing injuries and were not able to participate in the assessments; as such, 23 participants (18 ± 4 years, range 16–27 years) were included in the final analysis. Demographics and sport-related characteristics of the participants are reported in Table 1. The most prevalent reported injuries were in the right hamstrings (*n* = 4, 17.4%) and in both ankles (*n* = 5, 21.7%). TMG analysis showed that low lateral symmetry scores were mainly present in m.AL (*n* = 16, 69.6%), m.BF (*n* = 6, 26.1%), and m.TA (*n* = 9, 39.1%), while low functional symmetry scores were mainly present in the knees (right *n* = 8, 34.8%; left = 6, 26.1%) and ankles (right *n* = 12, 52.2%; left = 9, 39.1%).

### 3.1. CMJ Asymmetry, Bilateral Deficit, and TMG

**Figure 1:**
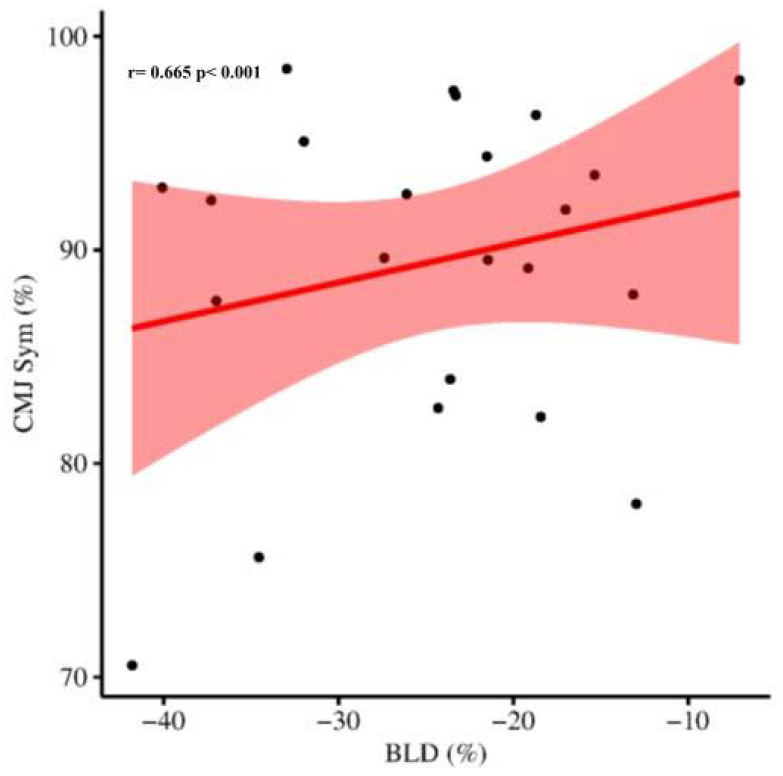
Correlation analysis between bilateral deficit (BLD, %) and countermovement jump (CMJ) inter-limb symmetry (CMJ Sym, %) in twenty-three male soccer players. Dots representing the individual data, fit line in red. Pearson’s coefficient r = 0.665, *p* < 0.001.

**Figure 2:**
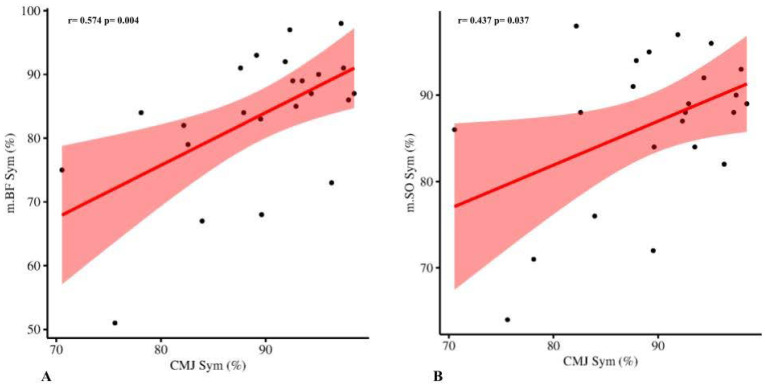
Correlation analysis between countermovement jump (CMJ) inter-limb symmetry (CMJ Sym, %) and (**A**) biceps femoris lateral symmetry (m.BF, %) Pearson’s coefficient r = 0.574, *p* = 0.004, and (**B**) biceps femoris lateral symmetry (m.BF, %) Pearson’s coefficient r = 0.437, *p* < 0.037 in twenty-three male soccer players. Dots representing the individual data, fit line in red.

Bilateral CMJ performance parameters (peak power and height) were found to significantly correlate with BLD (r = 0.682, *p* < 0.001), CMJ inter-limb symmetry (r = 0.488, *p* = 0.018), and m.GL (r = 0.486, *p* = 0.019) and m.VL (r = 0.418, *p* = 0.047) lateral symmetries. During CMJ, BLD was found to significantly correlate with CMJ inter-limb symmetry (r = 0.665, *p* < 0.001) (Figure 1), but none of the TMG asymmetries. Finally, CMJ inter-limb symmetry was found to significantly correlate with m.BF (r = 0.574, *p* = 0.004) and m.SO (r = 0.437, *p* = 0.037) lateral symmetries (Figure 2). Unilateral CMJ performance of the dominant limb showed significant correlations with m.AL Tc (r = −0.428, *p* = 0.042), m.GL Tc (r = −0.556, *p* = 0.006), and m.RF Tc (r = −0.532, *p* = 0.009), whereas on the non-dominant limb significant correlations were found between CMJ performance and m.GL Tc (r = −0.432, *p* = 0.039) and knee functional symmetries (r = 0.445, *p* = 0.033). CMJ and TMG outcomes are reported in Table 2.

### 3.2. Playing Position

**Figure 3:**
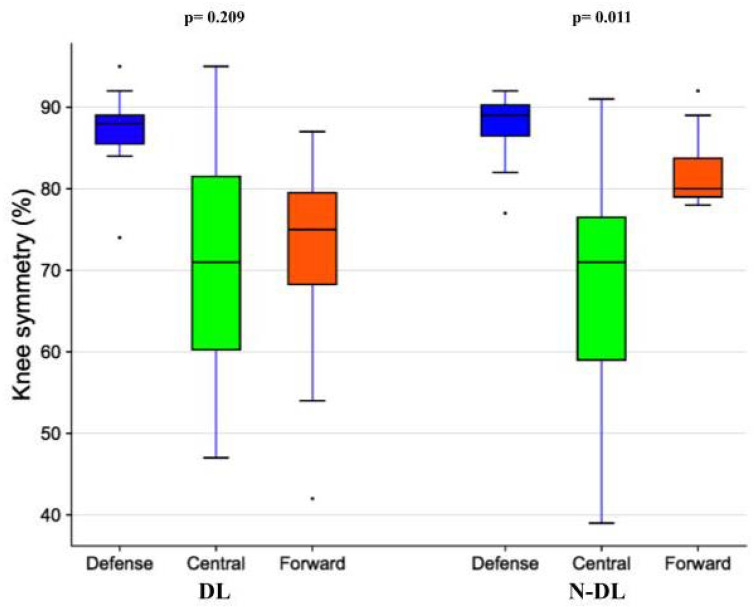
Boxplots representing the difference in the knee functional symmetry (%) of the dominant (DL) and non-dominant limb (N-DL) in 7 defense players, 8 central players, and 7 forward players. One-way analysis of variance (ANOVA).

None of the jump performance and symmetry parameters or TMG lateral symmetries were found to be significantly different between playing roles. Regarding TMG functional asymmetries, no side or side x position effects were found, whereas a significant playing position effect was found in the knee (F_2,19_ = 5.683, *p* = 0.012, pɳ2 = 0.374), with players in central roles presenting significantly worse symmetry scores than defense players (−17.5%, 95% CI −31.2–−3.9; *p* = 0.10), especially in the non-dominant limb (Figure 3). CMJ and TMG outcomes are reported in Table 3.

### 3.3. History of Injuries

**Figure 4:**
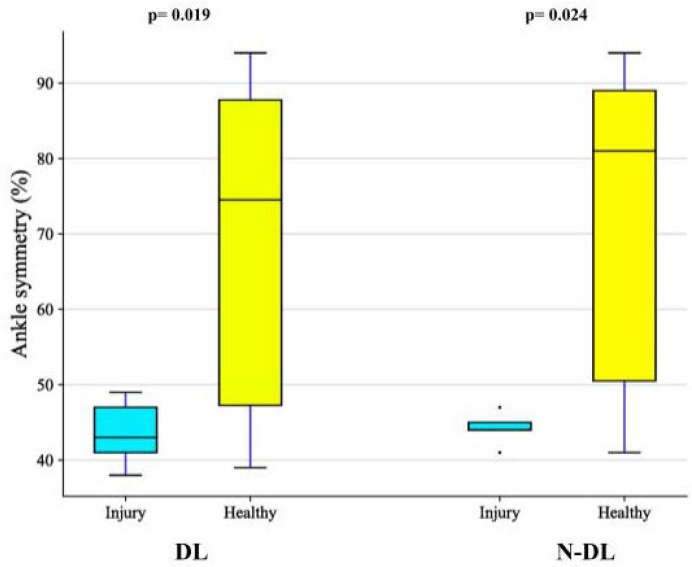
Boxplots representing the difference in the ankle functional symmetry (%) of the dominant (DL) and non-dominant limb (N-DL) in 5 players with a reported history of ankle injuries and 18 healthy players. Mann–Whitney U test.

Due to the limited prevalence of injuries among the included participants, TMG lateral and functional asymmetries were compared between those who reported a previous history of injuries at the ankles, finding significantly lower functional symmetry values in both the dominant (43%, 39.5–48.0 vs. 74.5%, 46.5–89.3, *p* = 0.019) and non-dominant (45%, 42.5–46.0 vs. 81.0%, 45.8–90.3, *p* = 0.024) limb (Figure 4). When a 65% cut-off value was used for ankle functional asymmetry, associations with previous ankle injuries were also found (*p* = 0.037 and *p* = 0.056, respectively).

## 4. Discussion

Prior studies have observed the presence of muscle asymmetries as possible factors associated with both performance and injury risk in sports. Our study has investigated the association between muscle asymmetries and bilateral deficit (BLD) during a countermovement jump (CMJ), and tensiomyography (TMG) parameters and asymmetries, in the lower limbs of male soccer players. The main findings from this study suggest that TMG evaluation of lower-limb muscle asymmetries may find some associations with CMJ power and height asymmetries, providing preliminary evidence of a muscular contractile component on a dynamic task such as a vertical jump.

Assessment of vertical jump performance is common in soccer, as it is an expression of lower-limb explosive strength and it is associated with competitive success [2]. In addition, it can provide an opportunity to assess and monitor mechanical inter-limb differences (i.e., asymmetries) [9], as well as bilateral deficits [12]. Vertical jump assessments, including CMJ, provide a physiological and biomechanical evaluation of a dynamic task, which can be influenced by several factors (including elastic properties, multi-joint movement, inter-and intramuscular coordination, and neuromuscular activation, etc.) [42]. TMG, in contrast, evaluates the contractile properties of skeletal muscles in a controlled condition, and only some muscles (those more superficial) can be assessed [43]. Despite such differences, results from this study provide preliminary evidence of potential associations between CMJ performance and TMG parameters, in particular as measures of lower-limb muscle asymmetry. CMJ inter-limb asymmetry was found to be correlated with lateral asymmetry of biceps femoris and soleus; such muscles could be implicated in vertical jump performance. In particular, these muscles may influence jump performance as a) early activation of the biceps femoris has been found to negatively influence the joint power transfer [44], reducing the effect of the stretch-shortening cycle, which is a key factor for performance in vertical jumps [45], and b) the soleus contributes to the center of mass (COM) acceleration during CMJ [46]. CMJ performance, in terms of bilateral or unilateral power and height, showed some association with gastrocnemius lateralis and vastus lateralis inter-limb asymmetry, and with adductor longus, gastrocnemius lateralis, and rectus femoris time to contraction (Tc). Since gastrocnemii and vasti muscles can influence COM acceleration, as previously discussed [46], it may therefore be speculated that asymmetries in such muscles may lead to unbalanced power production, and lower Tc values indicate an overall reduced explosive capacity of the muscle and lower expression of fast-contraction muscle fibers [47]. If rectus femoris and gastrocnemius lateralis contribution to vertical jump is expectable, adductor longus has a minimal contribution to force production in the sagittal plane, in which the countermovement jumps are performed [45]. However, this correlation is significant only during the unilateral CMJ, which in turn requires greater stabilization in which the adductor longus may participate, therefore suggesting a possible role for balance maintenance. The bilateral deficit was found to correlate only with CMJ inter-limb asymmetry, whereas none of the TMG showed any association. BLD has been suggested to be influenced by several factors, including population [36], task [48], joint angle [34], and contraction velocity [49], and it is an expression of altered muscle coordination [50]. Based on these assumptions, the absence of correlations between BLD and TMG might depend on the different characteristics of lower-limb muscle activation, i.e., a dynamic task during CMJ in contrast to a single muscle contraction during TMG.

Inconsistent results were present when the location of injuries and injuries rate were compared between playing positions; excluding goalkeepers, who usually present a lower risk of injuries, it seemed that no differences were present between forward, central, and defense players [51]. According to the present study, CMJ variables did not show significant differences between playing positions; previous research has suggested conflicting results, with some studies reporting forwards being faster and more explosive. Due to the limited sample size from this study, it is not possible to confirm the hypothesis that no differences are present in CMJ parameters in semi-professional young soccer players, although our findings are in line with some previous literature [52]. TMG was found to detect differences in muscle contractile properties between playing positions [32]. In the present results, players in the central roles were characterized by higher knee functional asymmetry compared with defense players. Despite the fact that such findings should be carefully considered due to the small sample size of the study, some hypotheses could be proposed considering previous findings suggesting different load and muscular responses between defense players and those playing in central or forward roles [53,54,55]), with midfielders sustaining the highest unavailability rates from a match and training injuries [56].

The identification of simple and easily accessible methods to detect risk factors for sport-related injuries can be of particular importance in promoting the implementation of strategies for the successful integration of evidence-based injury prevention programs into real-world soccer settings [57,58]. Muscle injuries can be among the major health issues faced by soccer players and are reported to represent up to 37% of all time-loss injuries at men’s professional level and up to 23% at men’s amateur level. Most injuries affect the lower extremities: the hamstring, adductor, quadriceps, and calf muscles are the most common injury locations. Almost all muscle injuries (>90%) occur in noncontact situations [59,60]; as such, the implementation of muscle-prevention exercises could help to reduce such injuries. For example, eccentric exercises (such as the “Nordic hamstring exercise”) [61], the FIFA 11+ protocol, balance training, and core stability exercises are all effective preventive interventions for hamstring strain injuries in soccer players [62]. 

There have been various studies in the literature that have tried to analyze the risk factors for muscle injuries. A history of groin injuries was found to led to a four-fold increase in future injuries in the same muscle [39]. The same evidence was found for calf strains [63] and for the four most commonly injured muscles in the lower extremity. In addition to this, previous injuries to other muscle groups were found to be a risk factor for other injuries in the lower extremities [60]. Age was also found to be a risk factor for muscle strain [60] because structural tissue changes are linked with progressive loss of important neuromuscular attributes such as power outputs or rate-of-force development and disruptions to motor unit discharge rates [63]. In addition, playing on natural grass [60], poor flexibility, lower levels of isometric adductor strength, and higher between-limb strength asymmetry [39] were determining factors for muscle injuries in the lower extremities. As such, the present results, combined with evidence from the previous literature, suggest that screening for muscle strength and asymmetry with easily accessible methods, such as vertical jump performance and TMG, could be of particular importance for the prevention of muscle injuries in men’s soccer. Based on these results and outcomes, specific muscle-strengthening exercises could be adopted in order to reduce lower extremities injuries.

Taken together, the findings from the present study, although preliminary and on a small sample, encourage the use of different evaluation techniques to detect muscle asymmetries in sports. Unfortunately, present results cannot confirm an association between BLD or jump performance asymmetries and previous injuries, whereas function asymmetry evaluated with TMG proposes some preliminary associations; nevertheless, longitudinal studies could better describe the role of muscle asymmetries as risk factors for future injuries, and the advantage of monitoring such asymmetries with jumping-based performance assessment that can be easily and quickly performed and interpreted, could be implemented in the field. 

### Limitations and Future Perspectives

This study included a sample of twenty-three young male soccer players, and, due to the interindividual differences, results should be cautiously interpreted and further studies on larger samples (with more athletes representative of the different playing positions) should be encouraged to confirm the observed associations. In addition, the integrated system to analyze CMJ outcomes only reported a limited set of outcomes and additional information might be useful, including eccentric and concentric forces. Only a few participants reported a previous history of lower-limb injuries, and TMG differences should be considered in light of such limitations. In addition, injuries were self-reported and more clinical information might help to describe the association between the injury and TMG asymmetries better. However, the presence of preliminary associations between ankle injuries and functional asymmetries involving the muscles acting on that specific joint encourage the use of TMG as a non-invasive tool that may help to detect agonist–antagonist unbalances, and therefore provide trainers and physiotherapists with some suggestions about the muscle groups that may benefit from dedicated strengthening or stretching protocols. Future studies should evaluate the presence of TMG-detected muscle asymmetries as predictors of ex novo or recurrence of injuries.

## 5. Conclusions

Results from the present study provide preliminary evidence of the association between countermovement jump and tensiomyography parameters, and, in particular, those outcomes evaluating inter-limb asymmetries. Inter-limb asymmetries of the lower limbs could be potential risk factors for sports-related injuries and should be monitored and properly evaluated to propose and implement prevention and training strategies. The combination of vertical jump and tensiomyography assessment could provide further useful information by describing different components (i.e., dynamic and “controlled” muscle contraction properties), which might participate in sport-specific tasks.

## Figures and Tables

**Table 1:** Demographics, training characteristics, health and injuries of the included participants. Medians (25th–75th percentile) and proportions, as appropriate.

	Participants (*n* = 23)
**Demographics**	
Age, y	18 ± 4
Body mass, kg	70.3 ± 5.4
Body height, m	1.80 ± 4.9
BMI, kg/m^2^	21.69 ± 1.44
**Training history and characteristics**	
Years of training in soccer, years	13 ± 5
Training frequency, training/week	3 ± 0.3
Training volume, min/week	318 ± 57
Strength & Conditioning, *n* (%)	20 (87.0)
Playing position, *n* (%)	
Goalkeeper	1 (4.3)
Defense	7 (30.4)
Center	8 (34.8)
Forward	7 (30.4)
**Health and injuries**	
Quadriceps, *n* (%)	
Right	1 (4.3)
Left	1 (4.3)
Hamstrings, *n* (%)	
Right	4 (17.4)
Left	1 (4.3)
Knee, *n* (%)	
Right	1 (4.3)
Left	2 (8.7)
Leg, *n* (%)	
Right	1 (4.3)
Left	2 (8.7)
Ankle, *n* (%)	
Right	5 (21.7)
Left	5 (21.7)

Notes: BMI: body mass index.

**Table 2:** CMJ performance and TMG parameters.

	Participants (*n* = 23)
**CMJ**	
Peak Power, W	
Bilateral	3870.7 ± 661.3
DL	2576.1 ± 485.4
N-DL	2602.3 ± 487.2
Inter-limb symmetry, %	89.4 ± 7.5
BLD, %	24.7 ± 9.4
Peak Height, cm	
Bilateral	45.4 ± 8.2
DL	34.0 ± 4.8
N-DL	35.1 ± 5.6
Inter-limb symmetry, %	91.9 ± 8.2
BLD, %	34.0 ± 10.6
**TMG**	
Lateral symmetry	
m.AL, %	67.3 ± 18.9
m.BF, %	83.5 ± 10.8
m.GL, %	87.4 ± 9.6
m.GM, %	89.0 ± 5.9
m.GT, %	85.6 ± 11.2
m.RF, %	87.0 ± 6.7
m.SO, %	86.7 ± 8.8
m.TA, %	77.1 ± 19.4
m.VL, %	88.7 ± 6.4
m.VM, %	88.3 ± 6.6
Functional symmetry	
Achilles tendon, %	
DL	87.4 ± 1.0
N-DL	85.1 ± 9.0
Ligament patellae, %	
DL	84.3 ± 6.7
N-DL	84.2 ± 8.5
Knee, %	
DL	74.7 ± 15.7
N-DL	77.9 ± 13.6
Ankle, %	
DL	63.3 ± 20.9
N-DL	66.5 ± 21.3
Leg, %	
DL	86.7 ± 7.1
N-DL	87.1 ± 7.5

Notes: countermovement jump (CMJ); dominant limb (DL); non-dominant limb (N-DL); bilateral deficit (BLD); tensiomyography (TMG); bilateral deficit (BLD). Muscles: adductor longus (m.AL), biceps femoris (m.BF), gastrocnemius lateralis (m.GL), gastrocnemius medialis (m.GM), gluteus major (m.GT), rectus femoris (m.RF), soleus (m.SO) tibialis anterior (m.TA), vastus lateralis (m.VL), vastus medialis (m.VM).

**Table 3:** CMJ performance and TMG parameters according to playing position.

	Defense	Central	Forward
	(*n* = 7)	(*n* = 8)	(*n* = 7)
**CMJ**			
Peak Power, W			
Bilateral	3902.3 ± 436.6	3631.5 ± 800.4	4043.0 ± 717.1
DL	2786.9 ± 556.1	2276.5 ± 431.8	2719.9 ± 374.5
N-DL	3033.1 ± 399.5	2377.4 ± 466.8	2421.3 ± 365.7
Inter-limb symmetry, %	87.5 ± 9.9	90.8 ± 5.5	89.2 ± 8.1
BLD, %	32.0 ± 9.8	22.4 ± 7.1	21.5 ± 8.4
Peak Height, cm			
Bilateral	44.9 ± 8.4	42.5 ± 7.3	50.3 ± 8.1
DL	36.6 ± 5.5	31.3 ± 4.6	35.1 ± 2.8
N-DL	38.6 ± 6.5	33.0 ± 4.5	35.0 ± 5.0
Inter-limb symmetry, %	89.5 ± 11.5	93.4 ± 7.8	92.3 ± 5.8
BLD, %	39.5 ± 13.4	33.8 ± 7.3	28.3 ± 9.4
**TMG**			
Lateral symmetry			
m.AL, %	64.3 ± 18.9	72.9 ± 14.6	62.7 ± 20.8
m.BF, %	82.9 ± 8.4	84.6 ± 11.1	82.1 ± 14.3
m.GL, %	89.1 ± 5.7	84.7 ± 15.4	88.6 ± 3.3
m.GM, %	86.1 ± 7.3	90.6 ± 5.4	90.0 ± 4.9
m.GT, %	90.2 ± 4.8	86.5 ± 7.8	80.3 ± 17.8
m.RF, %	86.1 ± 6.6	84.3 ± 7.1	90.7 ± 5.8
m.SO, %	86.6 ± 7.8	88.0 ± 6.7	85.7 ± 12.7
m.TA, %	72.1 ± 24.7	77.9 ± 20.0	81.3 ± 15.4
m.VL, %	89.1 ± 6.5	86.9 ± 4.5	92.3 ± 6.0
m.VM, %	90.6 ± 6.3	86.6 ± 6.1	86.6 ± 7.2
Functional symmetry			
Achilles tendon, %			
DL	86.6 ± 5.0	84.4 ± 15.0	91.1 ± 6.8
N-DL	78.5 ± 8.6	89.5 ± 9.5	86.4 ± 6.0
Ligament patellae, %			
DL	84.1 ± 6.5	84.4 ± 6.7	85.6 ± 7.5
N-DL	86.7 ± 5.1	83.1 ± 12.9	81.9 ± 4.7
Knee, %			
DL	86.7 ± 5.1	70.9 ± 16.0	70.4 ± 16.0
N-DL	86.7 ± 6.7	67.8 ± 16.4	82.7 ± 5.5
Ankle, %			
DL	59.0 ± 23.7	70.9 ± 21.3	55.0 ± 14.3
N-DL	73.4 ± 21.6	69.9 ± 22.8	53.7 ± 17.2
Leg, %			
DL	85.4 ± 8.0	86.8 ± 9.6	87.3 ± 3.0
N-DL	83.3 ± 11.6	89.1 ± 5.6	88.1 ± 2.8

Notes: countermovement jump (CMJ); dominant limb (DL); non-dominant limb (N-DL); bilateral deficit (BLD); tensiomyography (TMG); bilateral deficit (BLD). Muscles: adductor longus (m.AL), biceps femoris (m.BF), gastrocnemius lateralis (m.GL), gastrocnemius medialis (m.GM), gluteus major (m.GT), rectus femoris (m.RF), soleus (m.SO) tibialis anterior (m.TA), vastus lateralis (m.VL), vastus medialis (m.VM).

## Data Availability

Anonymized data are available upon reasonable request to the corresponding author according to the standard institutional procedure.
